# Epidemiology of preterm birth in Ethiopia: systematic review and meta-analysis

**DOI:** 10.1186/s12884-020-03271-6

**Published:** 2020-09-29

**Authors:** Kindie Fentahun Muchie, Ayenew Molla Lakew, Destaw Fetene Teshome, Melaku Kindie Yenit, Malede Mequanent Sisay, Fantahun Ayenew Mekonnen, Yohanes Ayanaw Habitu

**Affiliations:** 1 Department of Epidemiology and Biostatistics, School of Public Health, College of Medicine and Health Sciences, Bahir Dar University, Bahir Dar, Ethiopia; 2 Department of Epidemiology and Biostatistics, Institute of Public Health, College of Medicine and Health Sciences, University of Gondar, Gondar, Ethiopia; 3 Department of Reproductive Health, Institute of Public Health, College of Medicine and Health Sciences, University of Gondar, Gondar, Ethiopia

**Keywords:** Preterm birth, Prevalence, Associated factors, Ethiopia, Systematic review, Meta-analysis

## Abstract

**Background:**

Globally, complications of preterm birth are among the most common cause of neonatal mortality. In Ethiopia, the neonatal mortality reduction is not worthy of attention. Hence, this study reviewed the prevalence of preterm birth and factors associated with preterm birth in Ethiopia.

**Methods:**

The review protocol of this study has been registered in PROSPERO (CRD42017077356). The PRISMA guideline was followed for this review. Studies that assessed the prevalence and/or associated factors of preterm birth in Ethiopia and published from Jan 01, 2009 to Dec 31, 2019 were considered. Studies were searched from the PubMed and Science Direct among medical electronic databases and Google Scholar. Random-effects model was used for detected heterogeneity among studies. Publication bias and sensitivity analysis were assessed. Pooled estimates with its 95% confidence interval were reported using forest plots. The quality of evidence from the review was assessed using GRADE approach.

**Results:**

Twenty-two studies involving a total of 12,279 participants were included. The overall pooled prevalence of preterm birth in Ethiopia was 10.48% (95% CI: 7.98–12.99). Pooled odds ratio showed rural residence (AOR = 2.34, 95% CI: 1.35–4.05), being anemic (AOR = 2.59, 95% CI: 1.85–3.64), < 4 antenatal care visits (AOR = 2.34, 95%CI: 1.73–3.33), pregnancy induced hypertension (AOR = 3.49, 95% CI: 2.45–4.97), prelabor rapture of membrane (AOR = 4.42, 95% CI: 2.28–8.57), antepartum hemorrhage (AOR = 5.02, 95% CI: 2.90–8.68), multiple pregnancies (AOR = 3.89, 95% CI: 2.52–5.99), past adverse birth outcomes (AOR = 3.24, 95% CI: 2.53–4.15) and chronic illness (AOR = 4.89, 95%CI: 3.12–7.66) were associated with increased likelihood of preterm birth. Further, support during pregnancy was associated with reduced occurrence of preterm birth.

**Conclusion:**

The pooled national level prevalence of preterm birth in Ethiopia is high. Socio demographic, nutritional, health care, obstetric and gynecologic, chronic illness and medical conditions, behavioral and lifestyle factors are the major associated factors of preterm birth in Ethiopia. This evidence is graded as low grade. Thus, efforts should be intensified to address reported risk factors to relieve the burden of preterm birth in the study setting, Ethiopia.

## Background

According to the World Health Organization (WHO), preterm birth (PTB) is defined as a birth before 37 completed weeks of gestation [1]. It mostly occurs spontaneously for a variety of reasons [2]. Globally, 14.9 million PTBs were reported in 2010 resulting a birth rate of 11.1% (5–18%) [3]. The greater share (60%) of these PTBs were attributed by sub-Saharan Africa and South Asia countries where more than half (52%) of worldwide live births occurred. In 2014, the estimated worldwide PTB rate was 10·6% with 14·84 million live PTBs [4]. Asia and sub-Saharan Africa had showed an increasing trend that accounted for 12 million (81·1%) of the global PTBs [4].

Worldwide, complications of PTB are among the most common cause of neonatal and under-5 mortalities [5]. The risk of death due to its complication among under-5 children ranged from 1.9 to 155.1 per 1000 live births, in 2015 [5]. In addition, it results an apparent cost as the preterm neonates are the most admitted to neonatal intensive care unit (NICU) and with longer hospital stay globally [4]. Hence, PTB remains a crucial public issue in child mortality and in improving quality of maternal and newborn care [4].

Cognizant to the problem, the WHO is working to minimize public burdens like loss of lives and health problems arising from PTBs [6]. Currently, new guidelines including interventions provided to the newborn baby and the mother has been developed by WHO [6]. The movement, “Every Woman Every Child”, that puts the global strategy into action (2016–2030) aimed to ensure well-being of children, adolescents and women, and to end all preventable deaths within a generation [7]. It also envisioned to ensure that children, adolescents and women are at the heart of development by intensifying local and global commitment and action [7].

Ethiopia is one of the low-middle income countries in sub-Saharan Africa with high neonatal mortality [4]. Despite the fact that the country had achieved the millennium development goal-4 (reducing child and maternal mortality) with 67% under-five mortality reduction from the 1990 estimate, it is not worthy of attention [8]. To this effect, the country has further planned to reduce neonatal mortality rate from 28 death per 1000 live birth in 2015/16 to 10 deaths by 2019/2020 [9]. Furthermore, the country has also targeted to end preventable deaths of newborns and under-fives by 2030 [10].

A previous study on PTB conducted in Ethiopia assessed the association of pregnancy induced hypertension (PIH) and multiple pregnancies with PTB only [11]. Hence, there is a need for a comprehensive study that can aggregate previous studies to make it necessary for decision makers and implementers [12, 13]. Studies have demonstrated that PTB is affected by various factors. Therefore, this review aimed at assessing the pooled prevalence and associated factors of PTB in Ethiopia.

## Methods

### Development of the review method

The preferred reporting items for systematic reviews and meta-analyses (PRISMA) guideline was followed [14, 15]. The results of the review were reported according to the PRISMA guideline [16]. From the PRISMA flow chart [17], the four phases drawn were reported to show the overall process of study selection. The review protocol for this study has been registered in international prospective register of systematic reviews (PROSPERO) (CRD42017064585). The initial anticipated starting and completing dates were updated, where updates are not related to any problem to the present study. For further details, the readers can access the review protocol published in a peer reviewed open access journal [18].

### Eligibility criteria

All observational studies on PTB in Ethiopia were included. Studies that presented only qualitative results as well as case reports, editorials, review articles and case series on PTB were excluded. Only quantitative results were considered from studies that presented both qualitative and quantitative results. Studies that assessed the magnitude (prevalence) of and/or the associated factors of PTB were considered eligible. With regard to the magnitude, studies that had presented prevalence out of all live births were eligible. This review (systematic review and meta-analysis) study was conducted with the whole population of PTB (spontaneous or medically indicated). However, in the context of health care in the study setting, many deliveries are expected to be medically indicated.

### Search strategy

Literatures were searched from the medical electronic databases PubMed and Science Direct. Literatures were also hand-searched from Google Scholar. Furthermore, references list from included studies under critical review were also sought to retrieve additional studies. The literature search was limited to journal studies published from Jan 01, 2009 to Dec 31, 2019 in English. Medical Subject Headings (MeSH) terms from PubMed, and a combination of key words were applied to retrieve studies in the databases.

### Study selection process

The duplicated studies were excluded after retrieved studies were exported to the citation manager (EndNote) software. Based on the eligibility criteria, the titles and abstracts of the studies were reviewed for screening by the two authors (KFM and AML). Accordingly, studies were labeled as excluded, undecided or included. Considering the eligibility criteria, the full texts of the studies labeled undecided and included were reviewed independently for final inclusion by the two authors (KFM and AML). Ineligible studies were excluded by stating the reasons. Further, when authors found redundant publications with regard to prevalence and/or associated factors of PTB, the one that reported the results in an extractable form was included. Disagreements between the reviewers were addressed by consensus based discussion.

### Quality assessment

All of the eligible studies were appraised critically for their methodological robustness and validity. The three authors (DFT, MKY, FAM) appraised the studies using the Joanna Briggs Institute (JBI) Meta-Analysis of Statistics Assessment and Review Instrument [19]. The instrument has distinct checklist for various study designs. Disagreements among the reviewers were addressed by consensus based discussion and inviting another reviewer. Low risk (50%/above quality assessment score and 80%/above response rate) studies were included in the final review.

### Data extraction

The JBI data extraction form including characteristics of the study, definition of PTB, prevalence of PTB with its 95% confidence interval (CI), and list of associated factors with their effect size was used. The three reviewers (KFM, MMS and AML) extracted the information independently. Information from the extraction forms were transferred to a standardized excel sheet so as to make it ready for the systematic review. Disagreements among the reviewers were addressed by consensus based discussion.

### Data synthesis and statistical analysis

The summary of characteristics and the main findings of each included study were concisely presented using a table. The data were analyzed using Stata (version 14) software. A standard error (SE) for every study was computed from prevalence (p) and sample size (n) using the formula; SE = √(p x (1-p)/n). The overall national prevalence of PTB with its 95% CI was computed from SE and p of each study. Similarly, the effect measures of association between factors and PTB were computed using statistical estimates of odds ratio. Possible characteristics of the studies were sought as a basis for subgroup analysis.

The I^2^ statistics were used for assessing the level of heterogeneity among the studies [20]. A random effects meta-analysis model was conducted for the I^2^ value greater than 75%. Funnel plot and Egger’s statistical test were used to assess publication bias. Further, influential studies were assessed through sensitivity analysis.

The combined estimates with 95% CI were presented by forest plots. The GRADE approach was used to assess the quality of evidence. The narrative synthesis was performed for findings reported in only one study.

## Results

### Study selection

**Fig. 1:**
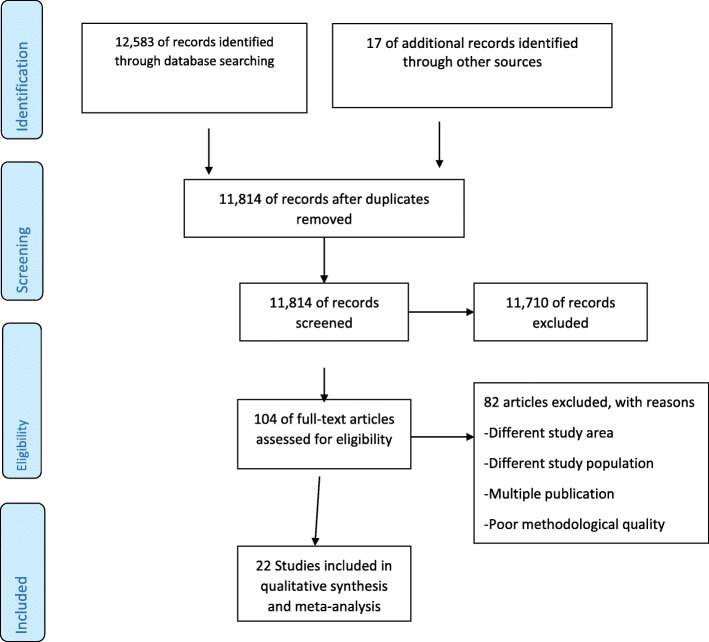
Flow chart of the review search results

A total of 12,600 research articles were retrieved, of which 11, 814 were retained after 786 were excluded due to duplication (Fig. 1). Detailed search engine and results are given in Table 1. By reviewing title and abstract, 104 articles were retained for full text review after excluding 11,710 ineligible studies. The remaining 104 full text articles were assessed for eligibility. Out of 104 studies, 81 were excluded with reason (different study area, different study population, or redundant publications [21, 22]) and 23 were retained for critical appraisal. Only one study [16] was excluded for its poor quality by critical appraisal. Finally, 22 studies [17, 19, 23–42] were included in this review.

**Table 1:** Search engine and results for systematic review and meta-analysis of PTB in Ethiopia

Source	Search Engine	# of research articles
PubMed	((((((incidence OR prevalence OR magnitude OR burden) AND Ethiopia AND (“Premature Birth/epidemiology”[Mesh] OR “Premature Birth/etiology”[Mesh] OR “Premature Birth/statistics and numerical data”[Mesh] OR premature birth* [MeSH] OR PTB OR premature birth OR preterm labo* OR preterm deliver* OR preterm infant OR preterm neonate* OR preterm newborn* OR birth outcome OR pregnancy outcome$ OR pregnancy complication$ OR birth outcome$ OR birth complication$)) AND (“2009/01/01”[PDat]: “3000/12/31”[PDat]) AND Humans [Mesh] AND English [lang])) OR (((“Premature Birth/epidemiology”[Mesh] OR “Premature Birth/etiology”[Mesh] OR “Premature Birth/statistics and numerical data”[Mesh] OR premature birth* [MeSH])) OR ((PTB OR premature birth OR preterm labo* OR preterm deliver* OR preterm infant OR preterm neonate* OR preterm newborn* OR birth outcome OR pregnancy outcome$ OR pregnancy complication$ OR birth outcome$ OR birth complication$) AND (predict* OR associated factor* OR risk factor* OR determinant*) AND Ethiopia) AND (“2009/01/01”[PDat]: “3000/12/31”[PDat]) AND Humans [Mesh] AND English [lang]))	10,783
Science Direct	((premature birth OR PTB OR pregnancy outcome OR birth complication) AND Ethiopia AND (incidence OR prevalence OR burden))	849
Science Direct	((premature birth OR PTB OR pregnancy outcome OR birth complication) AND Ethiopia AND (predictor OR associated factor OR risk factor OR determinant))	951
Google Scholar	A combination of the above key terms (preterm, premature, prevalence, proportion, magnitude, determinant, associated factor, factor, Ethiopia)	17

### Description of included studies

All twenty two studies included in this review had low risk quality (Additional file 1). The studies included in this review were conducted in Amhara, Oromia, Tigray and Southern Nations, Nationalities and Peoples (SNNP) regions. Of all the studies included five were case-controls, two were cohort and the remaining 15 were cross-sectional studies. Of these studies, 17 studies with a sample size of 10,389 and 13 studies were included respectively for assessing prevalence of and associated factors of PTB in Ethiopia. The highest prevalence of PTB was reported in Amhara (22.4%), whereas the lowest in Oromia (2.59%).

### Prevalence of PTB

**Fig. 2:**
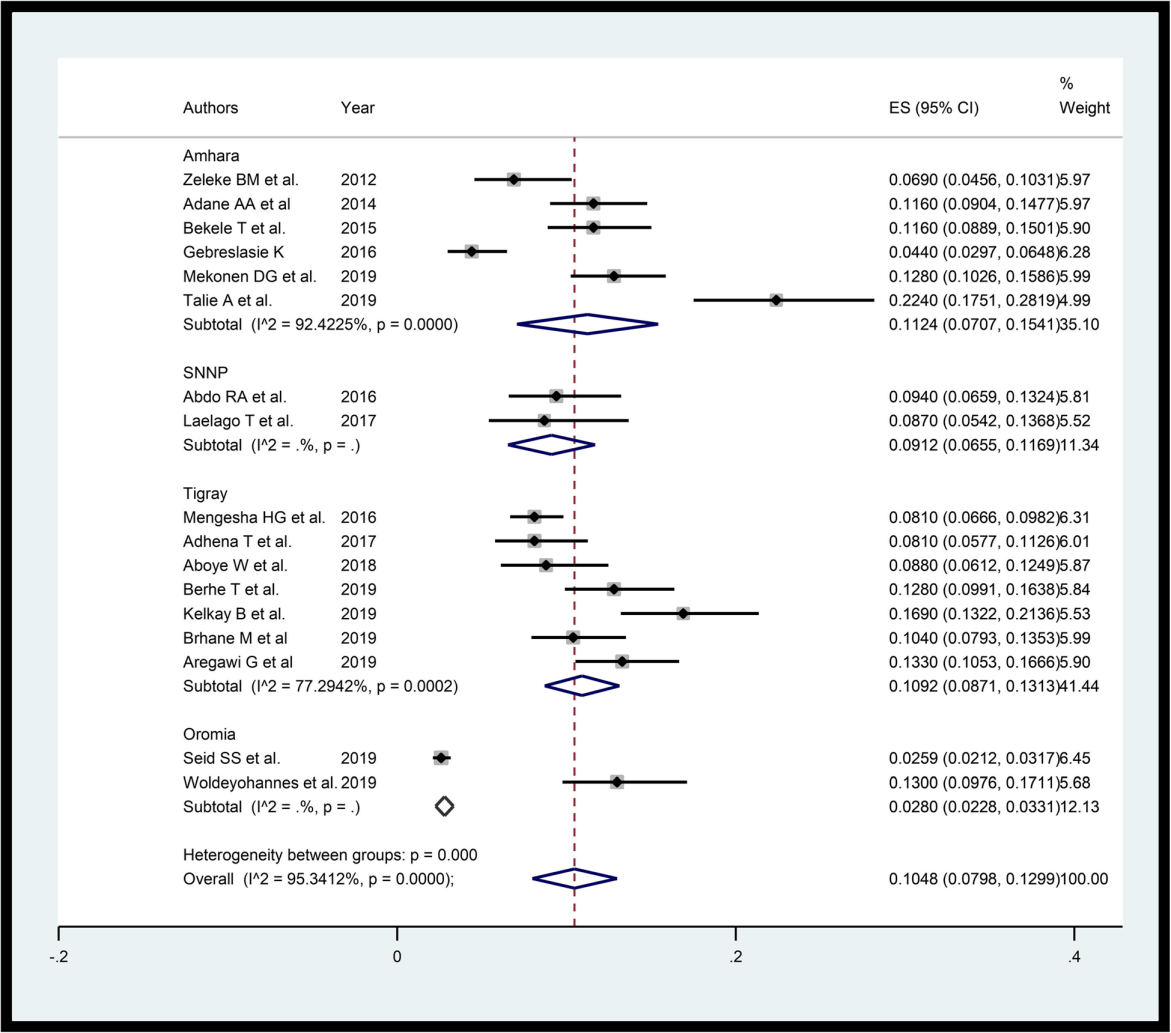
Forest plot displaying random-effects meta-analysis of the prevalence of PTB in Ethiopia

Funnel plot showed the existence of publication bias (Additional file 2). However, sensitivity analysis showed there is no influential study (Additional file 3). The random-effects meta-analysis using “metaprop” yielded that the overall pooled prevalence of PTB in Ethiopia was 10.48% (95% CI: 7.98–12.99) (Fig. 2). Sub-group analysis by region shows, 11.24% (95%CI: 7.07–15.41) in Amhara region, 10.92% (95% CI: 8.71–13.13) in Tigray region, 9.12% (6.55–11.69) in SNNP region and 2.8% (95% CI: 2.28–3.31) in Oromia region.

### Associated factors of PTB

The review of the associated factors are reported under five subtopics including socio demographic factors, nutritional and health care factors, obstetric and gynecologic factors, chronic illness and medical conditions, and behavioral and life style factors.

### Socio demographic factors

Using results from two studies [39, 42], the pooled effect shows that rural residents were 2.34 (AOR = 2.34, 95% CI: 1.35–4.05, I-squared = 0.0%) times likely to give birth before 37 week of gestation compared to urban residents. In contrast, one study shows that urban residents were more likely to give PTB [38]. Furthermore; lower income level [29, 37], illiterate educational status [42], household size of > = 4 [37], unmarried women [34], and lower maternal age (25–34 compared to ≥35 years) [42] had increased prevalence of delivering preterm.

### Nutritional and health care factors

Pooled effect of anemia was computed using results of five studies [24, 29, 33, 35, 39]. Egger’s small study bias test (*P*-value = 0.538) shows no serious small study publication bias. The pooled estimate (AOR = 2.59, 95% CI: 1.85–3.64, I-square = 54.1%) shows the existence of statistically significant association between iron deficiency anemia and PTB. Further, nutritional counseling [38] and folic acid supplementation [38] during pregnancy were associated with the reduced prevalence of PTB whereas the likelihood of giving PTB increased as severity status of maternal malnourishment increased [24, 34, 41].

Studies [24, 27, 34, 35, 37, 38] were pooled to assess the association between four or more antenatal care (ANC) visits and PTB. Insignificant (*P*-value = 0.238) Egger’s small study bias test shows no problem. The pooled estimate shows that those with < 4 ANC visits were 2.34 (AOR = 2.34, 95%CI: 1.73–3.33, I-squared = 54.7%) times likely to experience PTB compared to their counterparts. Similarly, having at least one ANC visit was associated with the reduced prevalence of PTB compared to no ANC visits at all [29]. Compared to having four or more ANC visits, having no ANC visits [42] and 1–4 ANC visits [33] had increased risk of delivering PTB.

### Obstetric and gynecological factors

A total of four studies [29, 33, 39, 42] with birth interval information were pooled. While pooling, Egger’s small study bias test and influential study analysis attested no problem. The estimated pooled values shows that those with less than 2 years birth interval were 2.91 (AOR = 2.91, 95%CI: 1.97–4.30, I-squared = 0.0%) times likely to deliver preterm compared to birth interval of more than or equal to 2 years.

Studies [19, 27, 31, 33, 39, 41, 42] were pooled to find the effect measures of association between pregnancy induced hypertension (PIH) and PTB. These studies have insignificant Egger’s statistical test (*p*-value = 0.601) showing there is no small study bias. The fixed effect pooled estimate shows that those who experienced PIH were 3.49 times likely (AOR = 3.49, 95%CI: 2.45–4.97, I-square = 40.6%) to have PTB.

Pooled estimate for association between prelabor rapture of membrane (PROM) and PTB used six studies [24, 27, 29, 33, 39, 41]. Since there was substantial heterogeneity, random effect estimate was considered. However, symmetric funnel plot and insignificant egger’s test (*p*-value = 0.191) shows there is no small study bias. Accordingly, a woman who experienced PROM for the current pregnancy was 4.42 (AOR = 4.42, 95%CI: 2.28–8.57, I-square = 76.0%) times likely to give PTB.

Three studies [33, 39, 41] were taken into account for pooled association regarding antepartum hemorrhage (APH). Egger’s test (*p*-value = 0.256) shows no problem of small study bias. The pooled estimate shows that those women with APH were 5.02 (AOR = 5.02, 95%CI: 2.90–8.68, I-square = 52.5%) times likely to have PTB.

To assess the pooled association of multiple pregnancy and PTB, three studies [27, 33, 39] with no small study bias were considered. Egger’s test (*p*-value = 0.895) shows no publication bias. The pooled result (AOR = 3.89, 95%CI: 2.52–5.99, I-square = 3.6%), shows multiple pregnancies were nearly four times likely to be delivered preterm.

Using two studies [27, 35], the pooled result (AOR = 4.12, 95%CI: 1.76–9.61, I-square = 20.1%) provides a woman with birth defect was 4.12 times likely to give PTB compared to their counterparts. Moreover; fetal distress [27], any gynecological problem such as problem of uterus and cervix [38] and preeclampsia [33] had increased the chance of preterm delivery.

**Fig. 3:**
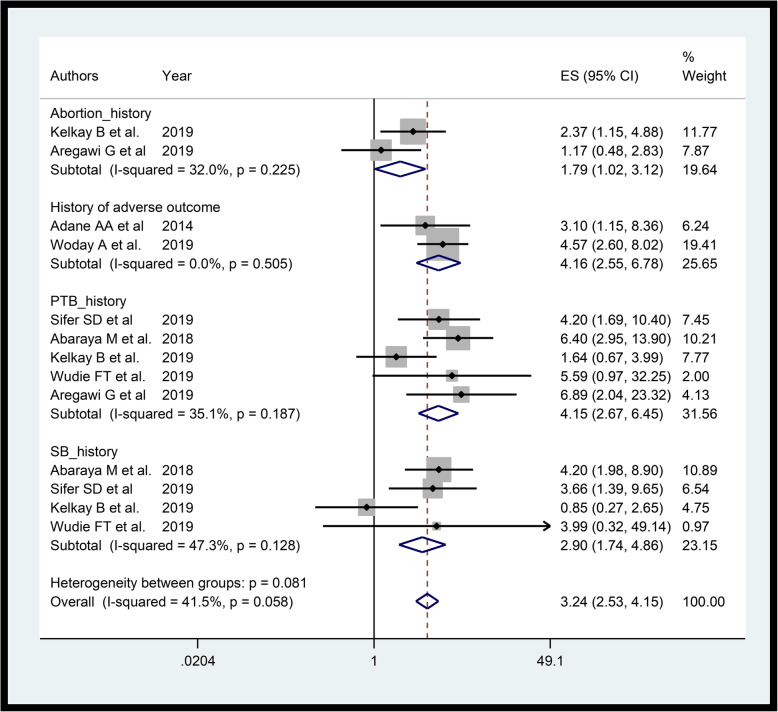
Forest plot displaying fixed effects meta-analysis of the association between past adverse birth outcomes and PTB in Ethiopia

We estimated pooled association between presence of past adverse outcomes and PTB using seven studies [19, 33, 35, 38, 39, 41, 42]. The funnel plot (Additional file 4) and Egger’s test (*p*-value = 0.692) shows there is no significant small study bias. The pooled estimate was 3.24 (AOR = 3.24, 95%CI: 2.53–4.15, I-square = 41.5%) (Fig. 3). Subgroup analysis by type of past adverse outcome shows, PTB (AOR = 4.15, 95%CI: 2.67–6.45, I-square = 35.1%), still birth (AOR = 2.90, 95%CI: 1.74–4.86, I-square = 47.3%) and, abortion (AOR = 1.79, 95%CI: 1.02–3.12, I-square = 32.0%) were associated with PTB. Furthermore, age of < 18 years at first delivery [38], lower number of pregnancies [38], induced onset of labor [39] and low birth weight [35] had showed increased likelihood of delivering PTB.

### Chronic illness and medical conditions

**Fig. 4:**
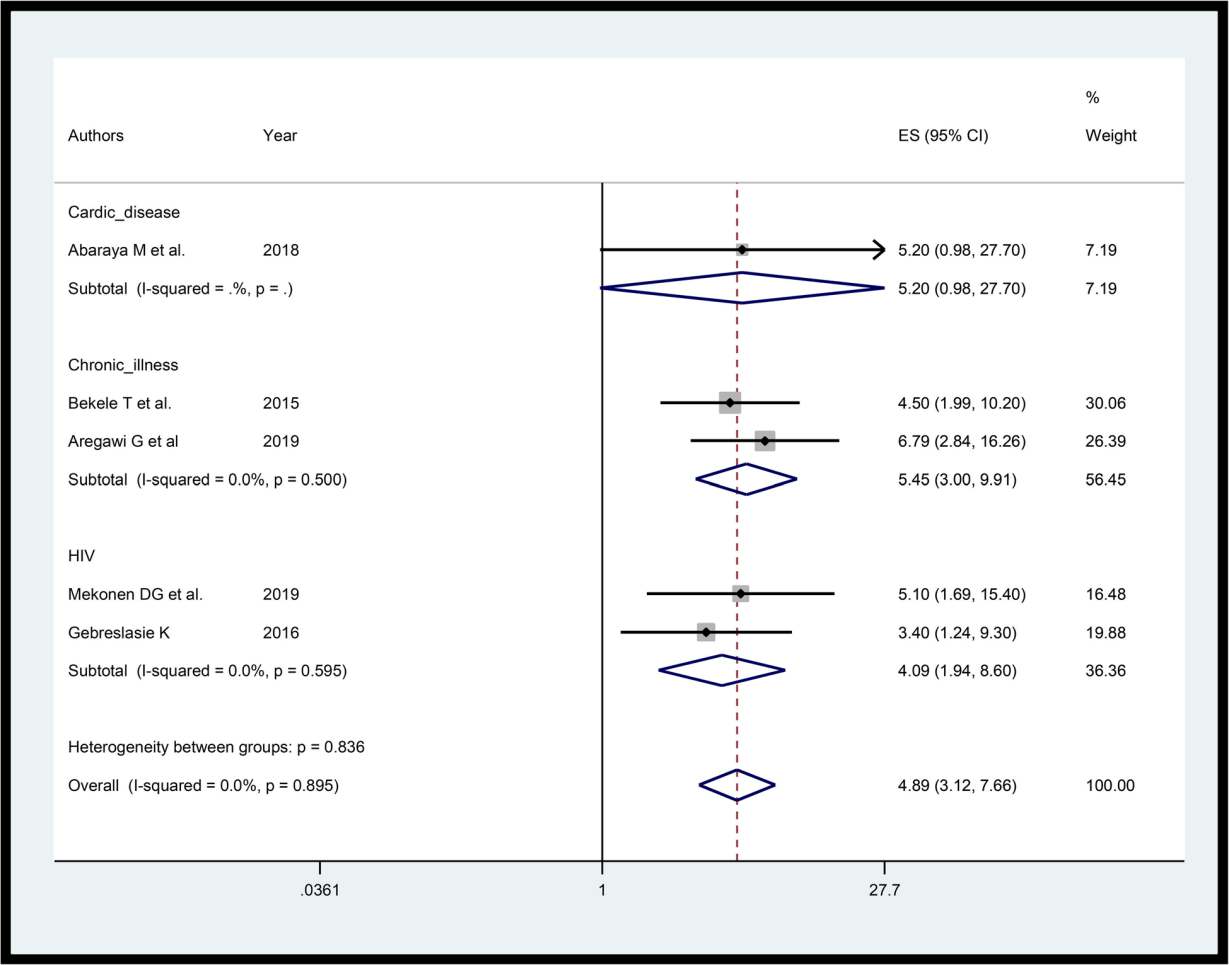
Forest plot showing the association of chronic diseases with PTB in Ethiopia

Studies [24, 29, 31, 33, 39] considered for pooling association of chronic diseases with preterm has no substantial heterogeneity and the funnel plot is also symmetric (Additional file 5). The pooled estimate shows those mothers with chronic illness were nearly four (AOR = 4.89, 95%CI: 3.12–7.66, I-squared = 0.0%) times likely to give PTB compared those free of chronic diseases (Fig. 4). Subgroup analysis by type of the disease (Cardiac disease, aggregate chronic disease and human immunodeficiency virus (HIV)) shows that likelihood of giving PTB among HIV carriers were more than fourfold (AOR = 4.09, 95%CI: 1.94–8.60, I-squared = 0.0%) compared to those not HIV carriers. Here “aggregate chronic disease” refers to any of chronic disease.

Regarding other medical conditions, the Egger’s small publication bias test (*p*-value = 0.098) and the funnel plot show no serious violation of the assumption. Accordingly, the pooled estimate (AOR = 3.78, 95%CI: 1.16–12.35, I-squared = 84.5%) [34, 39, 41, 42] shows presence of medical conditions like malaria and sexually transmitted infections (STI) increased the likelihood of PTB. Similarly, any of medical problem during pregnancy including Diabetes Mellitus (DM), Renal, or urinary tract infection (UTI) [42] were also positively associated with PTB.

### Behavioral and life style factors

Support during pregnancy reduced the chance of preterm delivery [38]. Contrarily; habit of standing for a long period of time [41], alcohol consumption [35, 42], cigarette smoking [35] and activity [38] during pregnancy were positively associated with PTB.

## Discussions

Ethiopia was one among the ten countries having highest number of PTBs and located in the region that contributed 81·1% of all PTBs globally in 2014 [4]. So, it is important to estimate the comprehensive burden of PTB and identify associated factors with its effect measures accounting the latest evidence in the country. Accordingly, country-wide burden of PTB was high and the associated factors were broadly identified as socio-demographic, nutritional, health care, obstetric and gynecologic, chronic illness and medical problems, and behavioral and life style.

The result shows that the overall prevalence of PTB in Ethiopia was 10.48%. This estimate is in line with global estimate in 2014 [4]. However, the estimate is greater than that of Kenya and Uganda in 2014 [4]. Furthermore, this result was higher than the prevalence of PTB in Iran from a review study [43, 44]. From the result there were inter-regional variation in prevalence of PTB with 11.24% in Amhara, 10.92% in Tigray, 9.12% in SNNP and 2.8% in Oromia. These show that there are no sufficient studies conducted in the remaining regions of the country. Though burden of PTB stratified by different gestational age thresholds like extremely preterm (< 28 weeks), very preterm (28–32 weeks) and moderate to late preterm (32–37 weeks) are very important [45], there were insufficient studies in the country that considered the stratification.

In the current review the pooled estimate shows being rural resident was associated with increased likelihood to give birth before 37 week of gestation. This is in line with another study conducted in China [46]. This might be due to easily accessible health facilities and better awareness in urban area than in rural area that could play substantial role in the prevention of preterm delivery. Further, rural resident women might be subjected to hard physical works that increase the chance of PTB. Unlike to the pooled result and the existing literatures, one study conducted in northern part of Ethiopia shows that urban women had increased likelihood of giving preterm birth [38]. This discrepancy might be due to the difference in the life style, awareness, maternal health care services accessibility and socioeconomic status.

The study reveals that maternal age of 25–34 years had increased risk of preterm birth. This finding is in line with finding of studies done in Kenya [47, 48] and Nigeria [49]. In contrast a longitudinal cohort study in Canada showed that reduced risk of preterm birth among mothers with age of 30–34 years [50]. This discrepancy might be explained by differences in clinical risk factors or socio-demographic factors among different studies.

Among nutritional factors; anemia, nutritional status, nutritional counseling and folic acid supplementation were associated with PTB. Anemia status was reported based on both hematocrit and hemoglobin levels of pregnant/mothers. In agreement with result of other studies [51, 52] anemic mothers are associated with increased likelihood of giving PTB. That might be because of anemia impairs the oxygen transportation, leading to placental insufficiency, what can finally result in PTB. Similarly, the result shows that likelihood of PTB increased as severity status of maternal malnourishment increased. A consistent result has been reported in different countries [53, 54]. This could be due to the direct effect of maternal nutritional status on placental size, strength of the membrane and fetus.

On the other hand, having nutritional counseling reduced the chance of having PTB. This might be due to the association between quality of diet during pregnancy and PTB [55]. Besides, our study shows that having folic acid supplementation reduced the chance of having PTB. Consistently, a similar study reported that Vitamin D insufficiency was associated with PTB [56]. This shows supplementary nutrients during pregnancy are crucial in low income countries like Ethiopia.

The pooled estimate shows that those with inadequate (< 4) ANC visits were more likely to give PTB compared to those with adequate ANC visits. It is consistent with results of other studies [47, 57, 58]. Likewise, having at least one ANC visit was associated with reduced chance of having PTB compared to no ANC visits. This is because of ANC service utilization offers a lot of information and care measures that may have role in improving health. In Ethiopia, inadequate ANC visits might happen because of scarcity of health care services and/or failure to seek health care.

The pooled value shows that those with birth interval of less than 2 years were 2.91 times likely to deliver preterm compared to more than or equal to 2 years. This is in line with other studies done [59–61]. This may be because mothers with short inter-pregnancy interval might not have adequate time to recover from their nutritional burden and physical stress of the pregnancy.

Those women who experienced PIH were more likely to have PTB. This result is in agreement with result of studies conducted in Ethiopia and other countries [58, 62, 63]. This might be due to reduction of the utero-placental blood floor by hypertension leading to intrauterine growth restriction finally causing preterm birth. Further, PIH could result placental vascular damage inducing the oxytocin receptors, which results in preterm labor and birth.

Women who experienced PROM for the current pregnancy were more likely to give PTB. This is in agreement with result of research and review articles [48, 64–67]. This might be due to activation of preterm labor as a response to raise of fetal interleukin-6 because of PROM [68]. Besides, in the majority of cases labor spontaneously initiates within a week and hours respectively after preterm and term PROM.

Besides, pooled association shows that women with APH were more likely to give PTB. This finding is consistent with the result of other studies [47, 48]. This might be due to the risk APH poses to a pregnant mother as well as the fetus. Similarly, the pooled result shows multiple pregnancies are more likely to be delivered preterm. The result is consistent with other reported studies [11, 58, 69–71]. This is due to overstretching of uterus might stimulate early labor.

The pooled result provides a woman with birth defect were 4.12 times likely to deliver preterm compared to their counterparts. Besides, preeclampsia had increased risk of PTB. Similar result has been reported by another study [58]. Moreover; fetal distress and gynecological problem had increased the chance of preterm delivery.

Women with past adverse outcomes were more likely to have PTB. Specifically, those with history of PTB, still birth, and abortion were more likely to give PTB. Consistently, studies reported that women with history of preterm birth, miscarriage and stillbirth had increased risk of preterm delivery [58, 72, 73]. Besides, related review studies [58, 61, 74] showed consistently that women with history of still birth were more likely to have PTB. Furthermore, in agreement with our study, other studies [49, 61, 75–77] showed those women with history of PTB were more likely to give PTB. Hence, special emphasis should be given for those women with history of adverse birth outcome.

The pooled estimate shows, those mothers with chronic illness were nearly four times more likely to give PTB. Subgroup analysis by type of the disease shows that likelihood of giving PTB among HIV carriers were more than fourfold compared to not HIV carriers. Consistent results were obtained from similar studies [78, 79]. This might be either chronic disease itself, or the medical treatment caused adverse reproductive outcomes.

The pooled estimate also shows presence of medical conditions like malaria and reactive STI increased the likelihood of PTB. Similarly, another study shows maternal malaria infection was positively associated with PTB [51]. Besides, a woman with urinary tract infections (UTIs) during pregnancy has increased chance of delivering preterm. In agreement with our study, women who had UTIs were more likely to have premature delivery [58]. This could be due to release of inflammatory mediators such as prostaglandins and matrix degrading enzymes triggered by infections stimulate uterine contraction.

Support during pregnancy had reduced the chance of preterm delivery. A similar study shows that women living without a partner had increased likelihood of preterm delivery [58]. This might be due to absence of someone to support during pregnancy may increase stress and thereby increase the likelihood of having preterm delivery. On the other way, we found out that a woman who consumes alcohol during pregnancy was more likely to give PTB. Consistently, a study reported that alcohol use during pregnancy increased the risk of preterm delivery [80].

Furthermore, habit of standing for long time and activity during pregnancy were positively associated with PTB. This might be due to the fact that standing for a long time increases the pressure on the blood vessels and leads difficulty of blood to return from the lower part of maternal body to heart. As a result congestion of blood in lower body can cause the uterus to contract regularly resulting in opening of cervix and preterm labor. Contrarily, result of other studies shows that higher leisure time physical activity [81] and aerobic exercise at early pregnancy for about 30–60 min for three to seven times per week [82] are associated with reduced risk of PTB among overweight and obese women. This difference might be due to study variations in the study population.

### Limitations

Inclusion of only quantitative observational studies published in English might have excluded those published in other languages and/or qualitative studies. Inclusion of most cross-sectional studies (15 out of 22) would be another possible limitation of the study in establishing cause effect relationship. In addition studies included lack uniformity in defining the population and estimation of gestational age. That is, most included studies did not explicitly mention whether or not only single and multiple pregnancies were considered in their studies. Moreover, lack of national birth registry in the country or regions of the country makes most PTB estimates rely on institutional reports that might not represent the whole population. Hence, the readers are suggested to take this into consideration.

## Conclusion

The prevalence of PTB in Ethiopia is high. Socio demographic, nutritional and health care, obstetric and gynecologic, chronic illness and medical conditions, and behavioral and lifestyle factors are the major associated factors of PTB in Ethiopia. Thus, efforts should be intensified to address the reported risk factors to alleviate the burden of PTB. This evidence is graded as low grade. There is inadequate number of studies in Ethiopia even those available are concentrated in only four regions (Tigray, Amhara, Oromia and SNNP regions). Hence further studies are recommended in the remaining regions of Ethiopia. Interventional studies are also recommended based on the identified factors so as to alleviate the problem.

## Supplementary information

**Additional file 1.** Characteristics of included studies in review of prevalence and determinants of PTB in Ethiopia.

**Additional file 2.** Funnel plot displaying a publication bias of the prevalence of PTB in Ethiopia.

**Additional file 3.** Sensitivity analysis displaying random effect for the prevalence of PTB in Ethiopia.

**Additional file 4.** Funnel plot for the association of past adverse birth outcome with PTB in Ethiopia.

**Additional file 5.** Funnel plot for association of chronic diseases with PTB in Ethiopia.

## Data Availability

The datasets analyzed during the current study are available from the corresponding author upon reasonable request.

## Abbreviations

ANC: Antenatal Care
AOR: Adjusted Odds Ratio
APH: Antepartum Hemorrhage
CI: Confidence Interval
DM: Diabetes Mellitus
HIV: Human Immunodeficiency Virus
JBI: Joanna Briggs Institute
MeSH: Medical Subject Headings
NICU: Neonatal Intensive Care Unit
PIH: Pregnancy Induced Hypertension
PRISMA: Preferred Reporting Items for Systematic Reviews and Meta-analyses
PROM: Prelabor Rapture of Membrane
PROSPERO: Prospective Register of Systematic Reviews
PTB: Preterm Birth
SE: Standard Error
SNNP: Southern Nations Nationalities and Peoples
STI: Sexually Transmitted Infection
UTI: Urinary Tract Infection
UTIs: Urinary Tract Infections
WHO: World Health Organization
