# Supplementary material for: Epidemiology of preterm birth in Ethiopia: systematic review and meta-analysis
Source: BMC Pregnancy Childbirth. 2020 Sep 29;20:574. doi: 10.1186/s12884-020-03271-6 (PMC7526155; doi:10.1186/s12884-020-03271-6)
Supplement: Supplementary file 1 — Additional file 1. Characteristics of included studies in review of prevalence and determinants of PTB in Ethiopia. [file 12884_2020_3271_MOESM1_ESM.docx]

| Authors | Year | Study Area | Region | Study Design | Sample size | PTB (%) | Quality status |
| --- | --- | --- | --- | --- | --- | --- | --- |
| Adane AA et al [19] | 2014 | Gondar | Amhara | Cross-sectional | 481 | 11.60 | Low risk |
| Mengesha HG et al. [25] | 2016 | Tigray Region | Tigray | Cohort | 1152 | 8.1 | Low risk |
| Laelago T et al. [23] | 2017 | Hadiya Zone, | SNNP | Cross-sectional | 183 | 8.7 | Low risk |
| Aboye W et al. [17] | 2018 | Aksum | Tigray | Cross-sectional | 308 | 8.8 | Low risk |
| Teklay G et al. [27] | 2018 | Central Tigray | Tigray | Case-control | 264 | NA | Low risk |
| Mekonen DG et al. [24] | 2019 | Debre Tabor | Amhara | Cross-sectional | 548 | 12.80 | Low risk |
| Talie A et al. [26] | 2019 | Awi Zone | Amhara | Cross-sectional | 232 | 22.4 | Low risk |
| Zeleke BM et al. [28] | 2012 | Gondar | Amhara | Cross-sectional | 305 | 6.9 | Low risk |
| Bekele T et al. [29] | 2015 | Debre Markos | Amhara | Cross-sectional | 422 | 11.6 | Low risk |
| Abdo RA et al. [30] | 2016 | Hadiya Zone | SNNP | Cross-sectional | 299 | 9.4 | Low risk |
| Gebreslasie K [31] | 2016 | Gondar | Amhara | Cross-sectional | 540 | 4.4 | Low risk |
| Adhena T et al. [32] | 2017 | Western Tigray | Tigray | Cross-sectional | 384 | 8.1 | Low risk |
| Abaraya m et al. [33] | 2018 | Jimma | Oromia | Case-control | 656 | NA | Low risk |
| Berhe T et al. [34] | 2019 | Central Tigray | Tigray | Cross-sectional | 410 | 12.8 | Low risk |
| Kelkay B et al. [35] | 2019 | Northwest Tigray | Tigray | Cross-sectional | 325 | 16.9 | Low risk |
| Seid SS et al. [36] | 2019 | Jimma | Oromia | Cross-sectional | 3546 | 2.59 | Low risk |
| Woldeyohannes et al. [37] | 2019 | Arsi Zone | Oromia | Cross-sectional | 322 | 13.0 | Low risk |
| Wudie FT et al. [38] | 2019 | Central Tigray | Tigray | Case-control | 288 | NA | Low risk |
| Aregawi G et al [39] | 2019 | Axum and Adwa | Tigray | Cross-sectional | 472 | 13.3 | Low risk |
| Brhane M et al [40] | 2019 | Northwest Zone | Tigray | Cohort | 460 | 10.4 | Low risk |
| Sifer SD et al [41] | 2019 | Sidama | SNNP | Case-control | 280 | NA | Low risk |
| Woday A et al. [42] | 2019 | Amhara Region | Amhara | Case-control | 402 | NA | Low risk |
