# Supplementary figures and images for: Epidemiology of preterm birth in Ethiopia: systematic review and meta-analysis
Source: BMC Pregnancy Childbirth. 2020 Sep 29;20:574. doi: 10.1186/s12884-020-03271-6 (PMC7526155; doi:10.1186/s12884-020-03271-6)

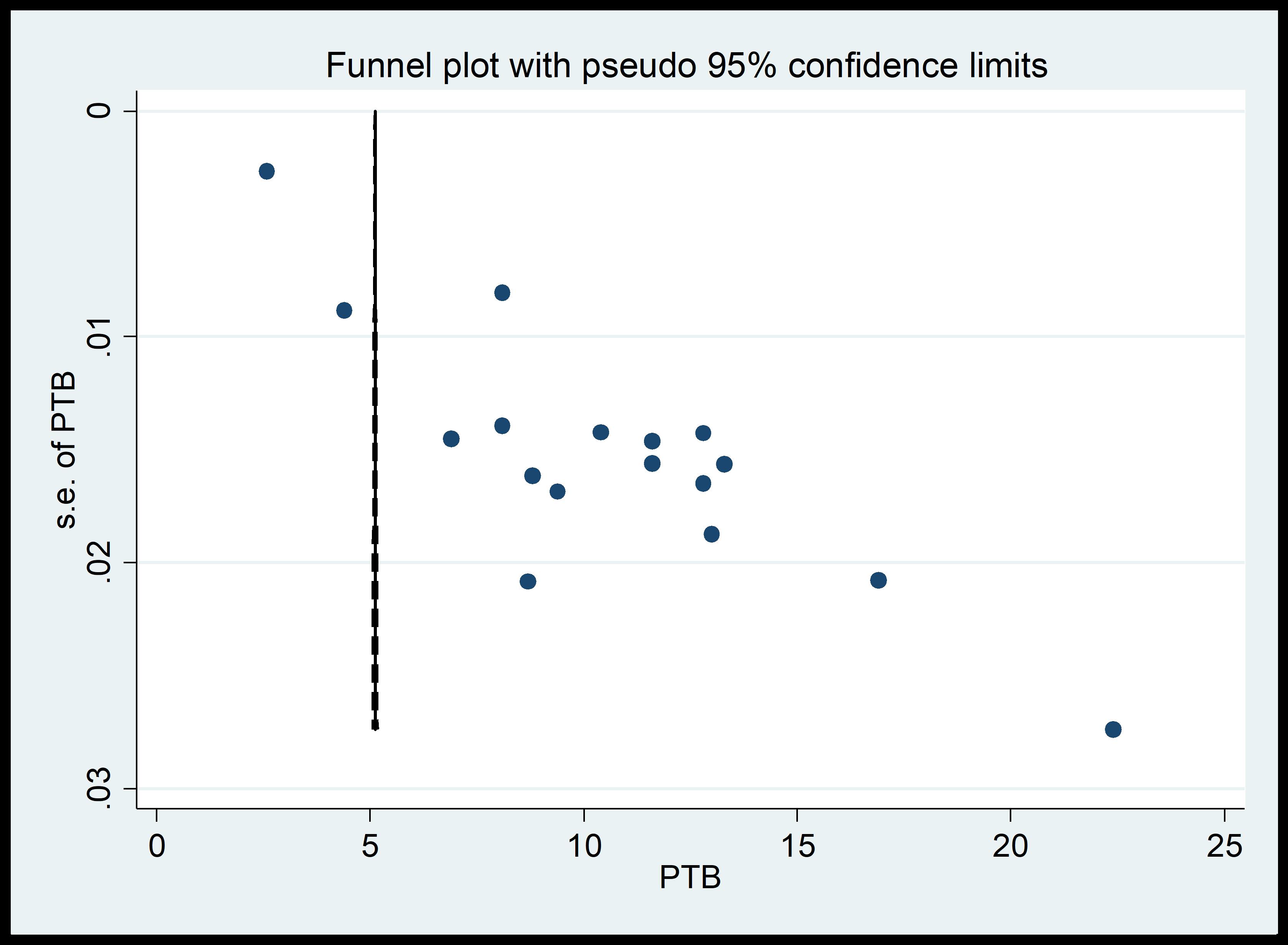

Supplement: Supplementary file 2 — Additional file 2. Funnel plot displaying a publication bias of the prevalence of PTB in Ethiopia. [file 12884_2020_3271_MOESM2_ESM.jpg]

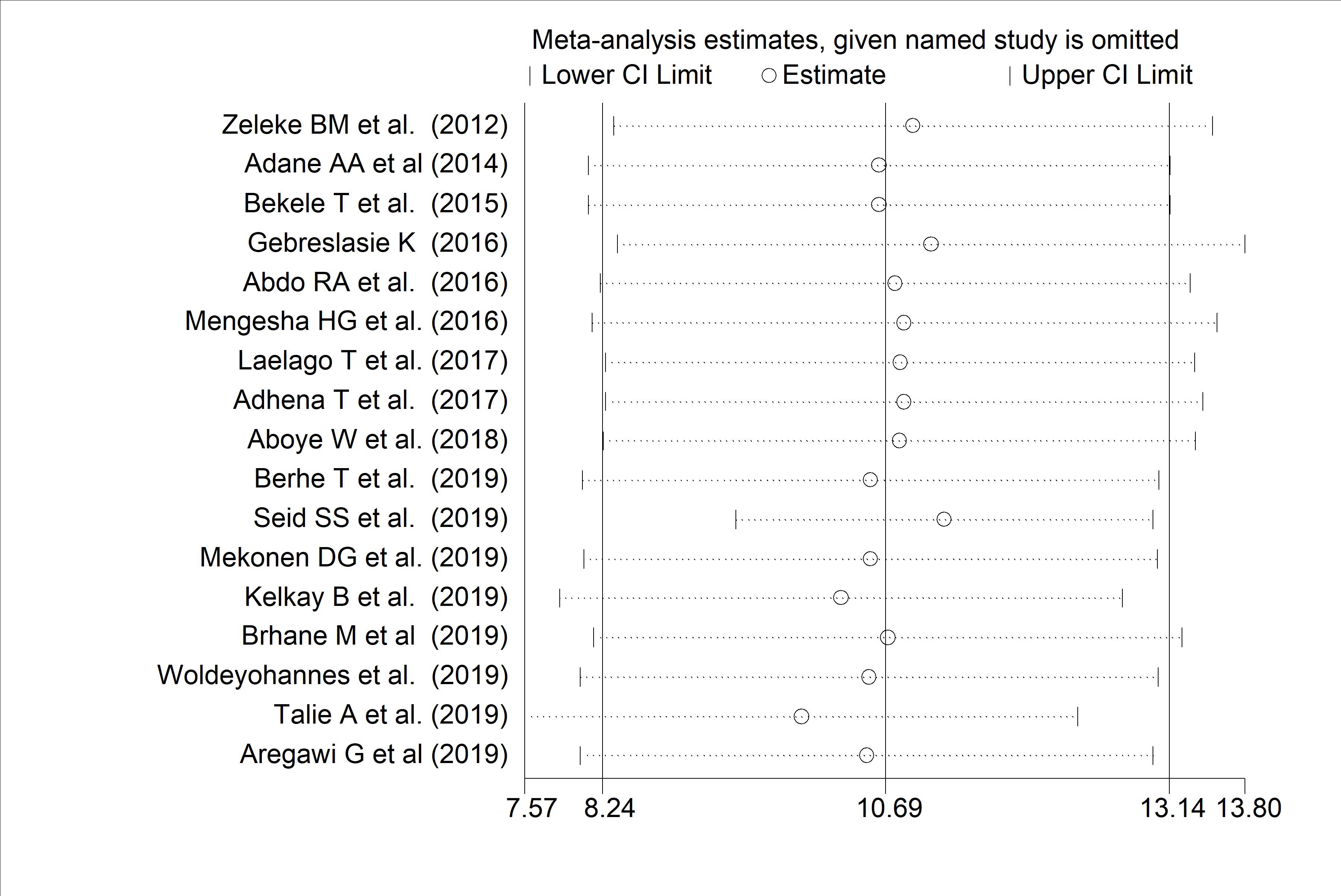

Supplement: Supplementary file 3 — Additional file 3. Sensitivity analysis displaying random effect for the prevalence of PTB in Ethiopia. [file 12884_2020_3271_MOESM3_ESM.jpg]

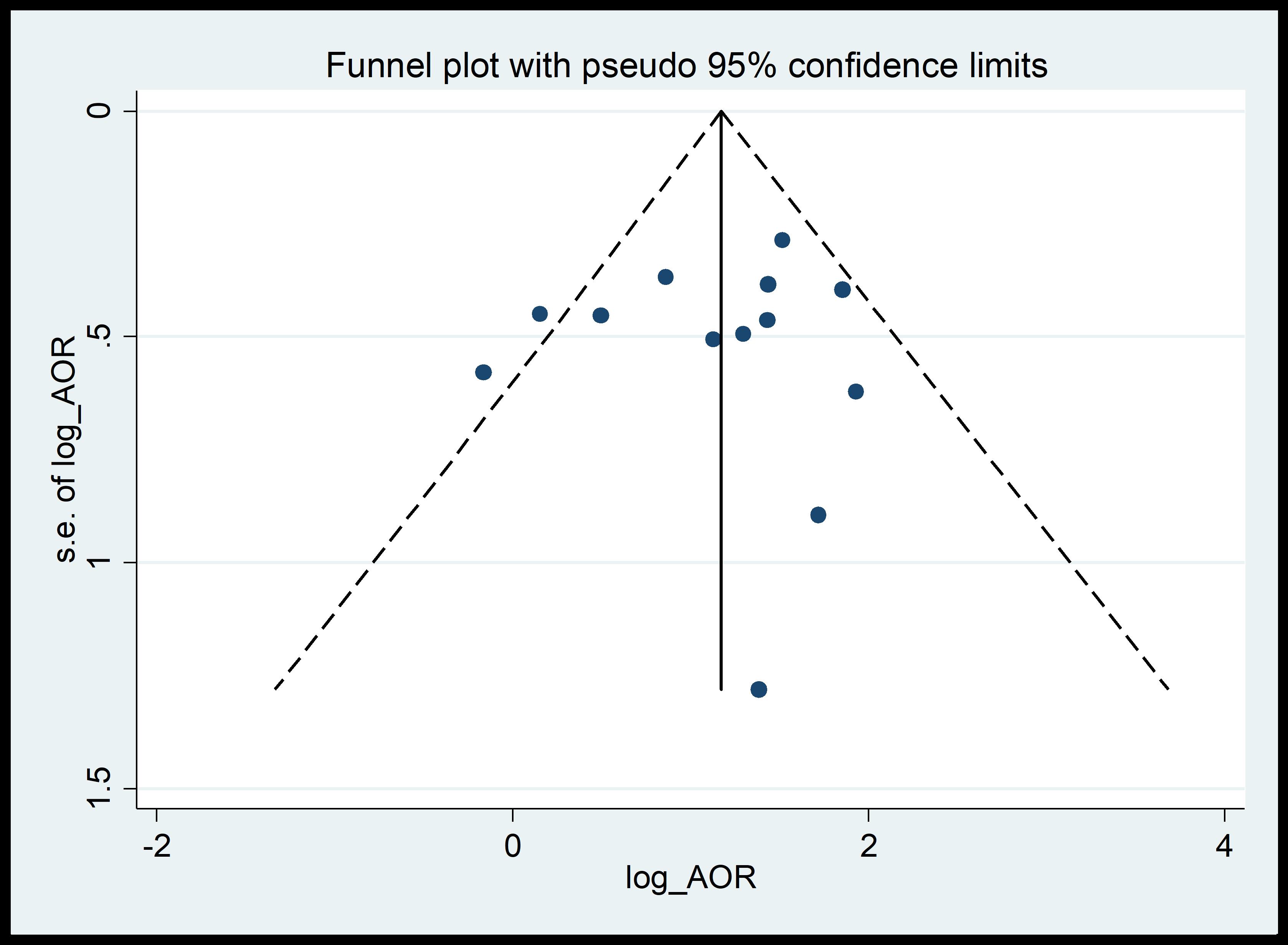

Supplement: Supplementary file 4 — Additional file 4. Funnel plot for the association of past adverse birth outcome with PTB in Ethiopia. [file 12884_2020_3271_MOESM4_ESM.jpg]

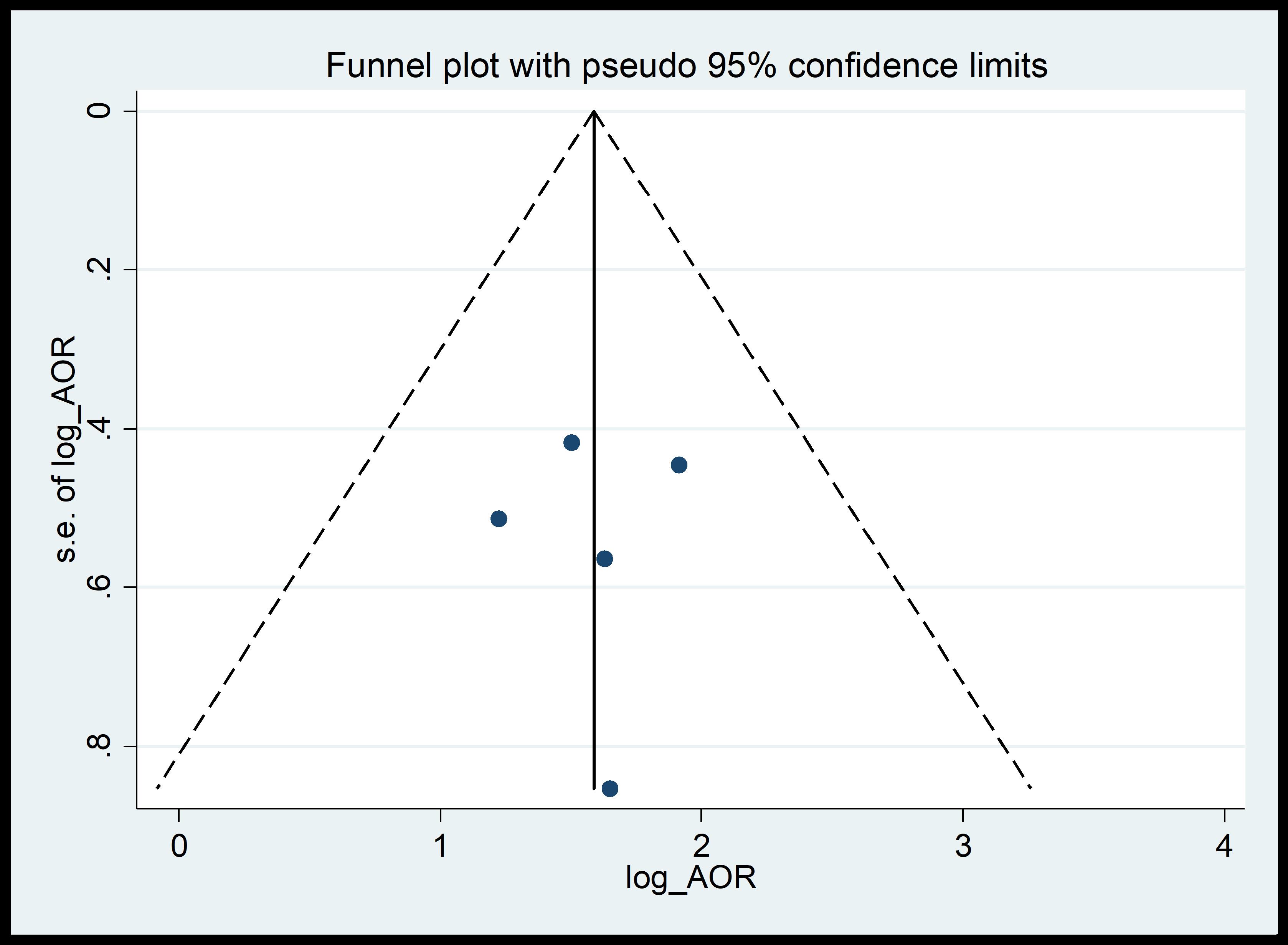

Supplement: Supplementary file 5 — Additional file 5. Funnel plot for association of chronic diseases with PTB in Ethiopia. [file 12884_2020_3271_MOESM5_ESM.jpg]
